# Inhaled GM-CSF administered during ongoing pneumovirus infection alters myeloid and CD8 T cell immunity without affecting disease outcome

**DOI:** 10.3389/fimmu.2024.1439789

**Published:** 2024-10-08

**Authors:** Nincy Debeuf, Julie Deckers, Sahine Lameire, Cedric Bosteels, Hamida Hammad, Bart N. Lambrecht

**Affiliations:** ^1^ Laboratory of Immunoregulation and Mucosal Immunology, VIB Center for Inflammation Research, Ghent, Belgium; ^2^ Department of Internal Medicine and Pediatrics, Ghent University, Ghent, Belgium; ^3^ Department of Pulmonary Medicine, Erasmus University Medical Center Rotterdam, Rotterdam, Netherlands

**Keywords:** GM-CSF, pneumonia virus of mice, viral infection, CD8 T cells, inhalation therapy

## Abstract

Granulocyte-macrophage colony stimulating factor (GM-CSF) is a pleiotropic cytokine, able to promote both myelopoiesis and activation of immune cells. Particularly in the lung, GM-CSF plays an important homeostatic role in the development and maintenance of alveolar macrophages, and is therefore considered to play a role in respiratory virus infections such as influenza and SARS-CoV-2, although the benefits of GM-CSF treatment in clinical studies remain inconclusive. To address this, we tested inhaled GM-CSF treatment in the Pneumonia Virus of Mice (PVM) mouse model. Our findings show that local GM-CSF therapy during PVM disease increased local neutrophilia and monocyte-derived cell influx, but diminished CD8^+^ T cells responses. Despite this, the observed effects on T cells and myeloid cells did not result in an altered clinical outcome during PVM infection. We conclude that inhaled GM-CSF therapy cannot be considered as a universal protective therapy in respiratory virus infections.

## Introduction

Granulocyte-macrophage colony stimulating factor (GM-CSF) is a hematopoietic cytokine that stimulates the differentiation of granulocytes, monocytes and macrophages from granulocyte-macrophage progenitors in the bone marrow, and their activation or “priming” in peripheral tissues. By stimulating various immune cells, and promoting emergency myelopoiesis, GM-CSF has also been recognized as an inflammatory cytokine in several immune disorders, such as asthma, rheumatoid arthritis and multiple sclerosis, where innate and adaptive lymphocytes as well as epithelial cells and fibroblasts appear as important cellular sources of the cytokine (1, 2). In some instances however, and particularly in the lung environment, GM-CSF has also been recognized for its anti-inflammatory homeostatic effects, since GM-CSF is required for the development and homeostasis of alveolar macrophages (AMs). AMs line the alveoli of the lungs and sample and subsequently conceal inhaled pathogens and particles, while preventing harmful inflammation in the delicate gas-exchange apparatus of the lungs (3). Furthermore, they also handle and recycle surfactant, a detergent that keeps alveoli in an open inflated state (4). Therefore, mice lacking GM-CSF or its receptor, and thus alveolar macrophages, develop pulmonary alveolar proteinosis (5, 6).

GM-CSF has been shown to play an important role during respiratory virus infections. In COVID-19 patients, inflammatory monocytes and macrophages replace the homeostatic AMs, which might be attributed to a lack of GM-CSF signaling (7). In an approach to restore the homeostatic AM pool, several studies have recently shown that inhaled GM-CSF can be safely used in COVID-19 (7–10). In a proof-of-concept trial, we have previously shown that inhaled GM-CSF led to an increased CD8^+^ T cell response in SARS-CoV-2 infected patients. In line with this, Barman et al. (8) demonstrated that inhaled GM-CSF improves survival in a mouse model of SARS-CoV-2/*S. pneumonia* co-infection (8). In mouse models of influenza, prophylactic intranasal GM-CSF treatment protects PR8-infected mice from weight loss and death by promoting the function of AMs and CD103^+^ dendritic cells (DCs) (11–13). Accordingly, conventional cDC1 dendritic cells in the lungs are dependent on GM-CSF for their homeostasis and the induction of CD8 T cell responses (14).

Although there is some evidence that local GM-CSF administration might be beneficial in the treatment and clearance of some respiratory virus infections, it is still unclear whether these effects are universal and can be extrapolated to other respiratory viruses, such as members of the pneumovirus family of viruses, like human Respiratory Syncitial Virus (hRSV), a virus that causes respiratory morbidity in the very young and the elderly frail, and in patients with underlying lung diseases. To address this, we tested inhaled GM-CSF treatment in the Pneumonia Virus of Mice (PVM) mouse model. PVM is the murine counterpart of hRSV and a natural pathogen of mice, requiring only a low inoculating infectious dose to lead to clinical manifestations, similar to hRSV pathology in humans. Our findings show that local GM-CSF therapy during PVM disease increased local neutrophilia and diminished rather than boosted CD8^+^ T cells responses, which did not benefit disease outcome. Thus, inhaled GM-CSF treatment is not universally protective in respiratory virus infections.

## Methods

### Mice

Female C57BL/6 mice were purchased from Janvier (France) and used between 7 to 12 weeks of age. All animals were housed under specific-pathogen-free conditions at the animal facility of Ghent University. Mice were housed in individually ventilated cages in a controlled day-night cycle and given food and water *ad libitum*. All experiments were approved by the animal ethics committee at Ghent University and were in accordance with Belgian animal protection law. PVM_M37-47 CD4^+^ and N339–347 specific CD8^+^ TCR transgenic (Tg) mice were generated in house, and were previously described (15, 16).

### PVM infection and *in vivo* GM-CSF administration

Mice were anesthetized via isoflurane (2 liters/min, 2 to 3%; Abbott Laboratories) and intratracheally infected with 50 pfu PVM (J3666 strain) or mock diluted in 80 µl PBS. Mice were monitored daily for weight, appearance and behavior. Recombinant murine GM-CSF (5 μg, VIB Protein Core Facility) was administered intranasally or intratracheally to anesthetized mice, diluted in 40 μl PBS or 80 μl PBS respectively.

### Flow cytometry

To measure inflammatory cell influx in the lung, mice were euthanized via an overdose pentobarbital. Bronchoalveolar lavage (BAL) fluid was obtained by flushing the lungs with EDTA-containing PBS (0.5 mmol/L). Lung lobes were isolated, cut with scissors and then digested for 30 minutes in RPMI-1640 (Gibco, Thermo Fisher Scientific) containing 20 μg/ml liberase TM (Roche), 10 U/ml DNase I (Roche), and 5% of FCS (Bodinco) at 37°C. Next, lungs were filtered through a 70 μm cell strainer. Lymph nodes were smashed in PBS through a 70 μm filter. Cell suspensions were stained with antibody cocktails in PBS for 30 minutes at 4°C and subsequently washed in PBS before readout. For tetramer staining, cells were incubated with MHCI (1:15 dilution) tetramer for 1 hour at room temperature and then stained for surface markers for 20 minutes at 4°C. PE-conjugated MHCI-N_339–347_ (GAPRNRELF) tetramer was purchased from BioLegend. To measure intracellular cytokines, cells were first restimulated for 4 hours with cell stimulation cocktail (eBioscience), and thereafter fixed and permeabilized using the BD Cytofix/Cytoperm kit (BD Biosciences) according to manufacturer’s instructions. Intracellular cytokine staining was performed overnight at 4°C. Unspecific antibody binding was prevented by adding 2.4G2 (antibody to the Fcg receptor II/III) during the staining. A fixed amount of counting beads (precision count beads; BioLegend) was added to determine absolute cell numbers. Dead cells were excluded by using the Fixable Viability Dye eFluor506 (eBioscience). Data acquisition was performed on a 5-laser Fortessa (BD Biosciences) and data were analyzed with FlowJo software (TreeStar, Ashland, Ore).

Lung single-cell suspensions were stained for flow cytometry using FITC-conjugated MHCII (M5/114.15.2; eBioscience), PerCP/Cy5.5-conjugated CD88 (20/70; BioLegend), eFluor450-conjugated Ly-6C (HK1.4; eBioscience), BV605-conjugated CD11b (M1/70; BioLegend), BV650-conjugated Ly-6G (1A8; BD Biosciences), BV711-conjugated CD64 (X54-5/7.1; BioLegend), BV786-conjugated Siglec-F (E50-2440; BD Biosciences), BUV395-conjugated CD4 (GK1.5; BD Biosciences), BUV496-conjugated CD8a (53-6.7; BD Biosciences), BUV805-conjugated CD45 (30-F11; BD Biosciences), APC-efluor780-conjugated TCR-β (H57-597; BioLegend), PE-efluor610-conjugated CD11c (N418; eBioscience), PE-Cy5-conjugated NK1.1 (PK136; BioLegend), PE-Cy5-conjugated CD19 (1D3; Thermo Fisher Scientific), AF647-conjugated CXCL9 (MIG-2F.5; BioLegend), PE-Cy7-conjugated IFN-γ (XMG1.2; eBioscience). To stain for DC subtypes, following antibodies were used: FITC-conjugated CD26 (H194-112; BioLegend), PerCP/Cy5.5-conjugated CD172a (P84; eBioscience), eFluor450-conjugated CD86 (GL1; BioLegend), BV605-conjugated CD11c (N418; BioLegend), BV650-conjugated XCR1 (ZET; BioLegend), BV711-conjugated CD88 (20/70; BD Biosciences), APC-conjugated CD40 (3/23; BD Biosciences), AF700-conjugated CD45 (30-F11; BioLegend), APC-eFluor780-conjugated MHCII (M5/114.15.2; eBioscience), PE-conjugated CD80 (16-10A1; BD Biosciences), biotin-conjugated FcERI (MAR-I; Life Technologies), PE-CF594-conjugated streptavidin (BD Biosciences), PE-Cy5-conjugated NK1.1 (PK136; BioLegend), PE-Cy5-conjugated CD19 (1D3; Thermo Fisher Scientific), PE-Cy5-conjugated CD3 (145-2c11; BioLegend), PE-Cy7 conjugated CCR7 (4B12; BioLegend) and BUV395-conjugated PD-L1 (TY25; BD Biosciences).

Lymph node single-cell suspensions were stained for flow cytometry using FITC-labelled CD8a (53-6.7; BioLegend), BB700-labelled CD69 (H1.2F3; BD Biosciences), V510-labelled TCR-β (H57-597; BioLegend), BV605-labelled CD45.1 (A20; BioLegend), BV786-labelled CD5 (53-7.3; BD Biosciences), RedFluor710-labelled CD44 (IM7; Tonbo Biosciences), PE-labelled CD62L (MEL-14; BD Biosciences), PE-Cy7-labelled IFN-γ (XMG1.2; eBioscience), BUV395-labelled CD4 (GK1.5; BD Biosciences) and BUV737-labelled CD45.2 (104; BD Biosciences). Proliferation was measured by using Cell Tracer Violet (CTV; eBioscience).

### Cytokine ELISA

To measure IL-6, IFN-γ and CCL-17, an eBioScience cytokine ELISA was performed according to manufacturer’s conditions.

### Histology

After overnight fixation in 4% paraformaldehyde solution, tissues were embedded in paraffin, cut into 5-μm slices, and stained with H&E. Images were taken with an AxioScan Slidescanner (Zeiss).

### T-cell adoptive transfer

PVM TCR Tg T cells were isolated out of spleens and lymph nodes from PVM TCR CD4 or CD8 Tg mice. T cells were purified by making use of the MagniSort Mouse CD4/8 T cell Enrichment Kit (Thermo Fisher) and were labelled with Cell Trace Violet (eBioscience) during 10 minutes at 37°C. CD45.1 TCR Tg T cells (1 × 10^6^) of each genotype were intravenously injected into CD45.2 C57BL/6 acceptor mice, followed by intratracheal infection with 35 pfu PVM 2 hours after intravenous injection. Acceptor mice were sacrificed 3 days later, and mediastinal lymph nodes, lungs, and spleens were dissected for analysis.

### Quantitative real time-PCR

Total RNA was isolated from mouse lung tissues by using TriPure Isolation Reagent (Roche) and isolated according to the manufacturer’s instructions. One microgram of RNA was converted to cDNA by using an iScript advanced reverse transcriptase (Bio-Rad Laboratories, Hercules, Calif), according to the manufacturer’s instructions. Expression levels were calculated by using qBase+ software (Biogazelle, Ghent, Belgium) and normalized to the 2 most stable reference genes among *Sdha*, *Hprt*, *Rpl13a*, and *Gapdh*. To amplify genes, SensiFAST SYBR No-ROX Kit (GC Biotech, Waddinxveen, the Netherlands) was used, and primers were as follows:

*PVM_SH_* (forward, AACAACAGCTAGGGTGGCTC; reverse, TCACCTGCGTCAGATAGGGA); *Sdha* (forward, TTTCAGAGACGGCCATGATCT; reverse, TGGGAATCCCACCCATGTT); *Hprt* (forward, TGAAGAGCTACTGTAATGATCAGTCAAC; reverse, AGCAAGCTTGCAACCTTAACCA); *Rpl13a* (forward; CCTGCTGCTCTCAAGGTTGTT; reverse, TGGTTGTCACTGCCTGGTACTT) and *Gapdh* (forward, GCATGGCCTTCCGTGTTC; reverse, TGTCATCATACTTGGCAGGTTTCT).

### Statistical analysis

Data were analyzed with a Shapiro-Wilk normality test to assess whether the data were normally distributed. Parametric data were analyzed with an ordinary one-way ANOVA test with multiple comparison correction. Nonparametric data were analyzed with an unpaired Kruskal-Wallis test with multiple correction. Data are shown as means ± SEMs. *P <.05, **P <.01, ***P <.001 and ****P<.0001.

## Results

### PVM infection is characterized by loss of AMs and an influx of neutrophils and CD8^+^ T cells in the alveolar space

**Figure 1:**
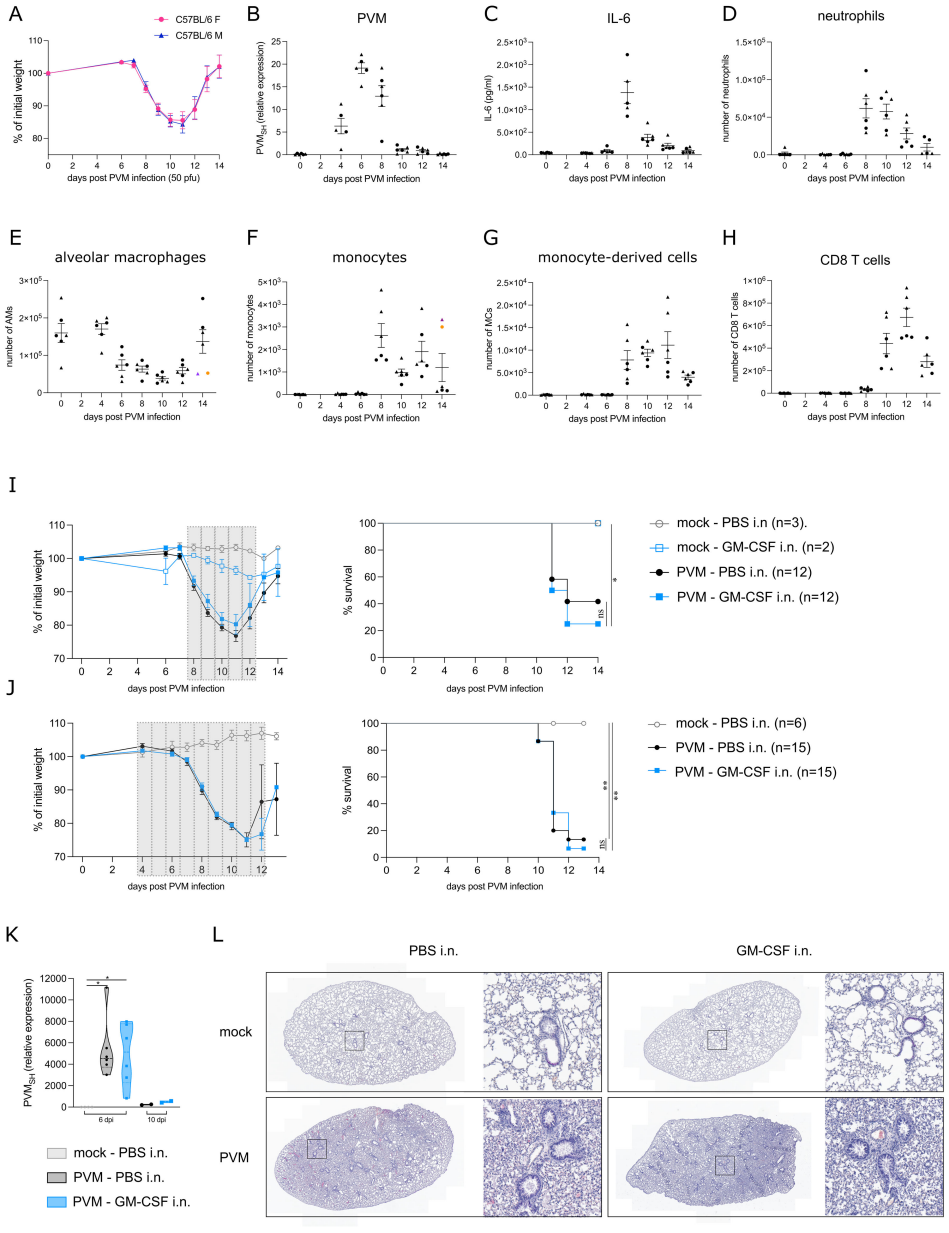
**(A)** Weight loss in C57BL/6 females (F) and males (M) upon PVM-infection with an LD50 dose of 50 pfu. **(B)** Expression of viral *PVM SH* gene relative to *hprt* and *rpl13a* as determined by qRT-PCR on lung tissue. Circles represent female mice, whereas triangles represent male mice. **(C)** IL-6 cytokine levels in BAL fluid. **(D-H)** Number of neutrophils, alveolar macrophages (AMs), monocytes, monocyte-derived cells (MCs) and CD8 T cells in BAL fluid. In **(E, F)**, colored symbols represent the same mouse. **(I, J)** Weight loss and Kaplan-Meier survival curves. The grey bars represent the days of GM-CSF treatment. Statistical analysis was performed by a Log-rank, Mantel-cox test. **(K)** Expression of viral *PVM SH* gene relative to the mock WT group as determined by qRT-PCR on lung tissue from 6 dpi and 10 dpi [experimental model as shown in **(J)**]. *Rpl13a* was used as housekeeping gene. **(L)** Representative H&E-stained lung sections of mock/PVM-infected mice upon PBS or GM-CSF intranasal treatment (11 dpi). ns = non-significant, *P < .05, **P < .01.

We first followed clinical symptoms and surrogate biomarkers of severity in C57BL/6 mice infected with PVM infection. Onset of symptoms upon PVM infection is a relatively late event, and associated with weight loss which is an often used clinical marker of disease severity. Upon intratracheal infection with an LD50 dose of 50 pfu PVM, mice started losing weight between 7 and 8 days post infection (dpi) (**Figure 1A**) without sex bias since male and female mice had the same degree of weight loss, with similar kinetics of the response. The viral titer peaked at 6 dpi, just before the onset of inflammation and weight loss (**Figure 1B**). Inflammatory cytokines, such as IL-6 have been used as biomarkers of disease severity in COVID-19, and in this PVM model peaked at 8 dpi in BAL fluid (**Figure 1C**). We next analyzed inflammatory cell influx at various days post infection. Upon analysis of immune cells in BAL fluid, neutrophils were detected as the major immune cell population recruited during the early inflammatory phase (**Figure 1D**). On the other hand, AMs gradually disappeared from the BAL fluid after PVM infection (**Figure 1E**), whereafter monocytes (Ly6C^hi^ CD88^+^ CD11b^+^ cells) and monocyte-derived cells (MCs) (characterized as CD11c^+^, MHCII^+^, CD64^+^ and CD88^+^ cells) replenished the myeloid cell pool in the BAL fluid (**Figures 1F, G**). CD8^+^ T cells mainly populated the alveolar space during the late inflammatory phase following PVM infection (**Figure 1H**).

### Local GM-CSF is not protective during PVM infection

The observed AM disappearance reaction upon PVM infection made us wonder whether this was due to lack of GM-CSF signaling, similarly to what has been observed in SARS-CoV-2 infected individuals (7). Therefore, we explored whether GM-CSF administration could have a therapeutic effect during PVM infection. Of note, GM-CSF was administered locally in the lungs, because systemic instillation of GM-CSF might induce emergency myelopoiesis and promote inflammation through priming of neutrophils (1, 2). To determine the ideal timing of treatment, several treatment regimens were explored, of which two representative experiments are shown in **Figures 1I, J**. In the upper panel, mice received 5 μg GM-CSF intranasally for 5 consecutive days from 8 dpi onwards, the time of maximal weight loss and clinical severity (**Figure 1I**). At that timepoint, AMs had significantly diminished and were substituted by inflammatory cells. In **Figure 1J**, intranasal GM-CSF treatment began at 4 dpi, preceding the onset of inflammation and the loss of AMs. However, in all these experiments, there was no beneficial effect of intranasal GM-CSF on PVM-induced weight loss and survival. Both in GM-CSF and PBS-treated mice, PVM virus was cleared by 10 dpi (**Figure 1K**). Furthermore, upon early GM-CSF treatment as in **Figure 1J**, viral load was similar between the two treatment groups at 6 dpi. Histological H&E analyses also revealed no differences between PBS and GM-CSF treated mice at 11 dpi, the timepoint with maximal weight loss. In both conditions, lungs were heavily inflamed compared to mock-treated mice (**Figure 1L**).

### Local GM-CSF changes the immune cell composition in the lung

**Figure 2:**
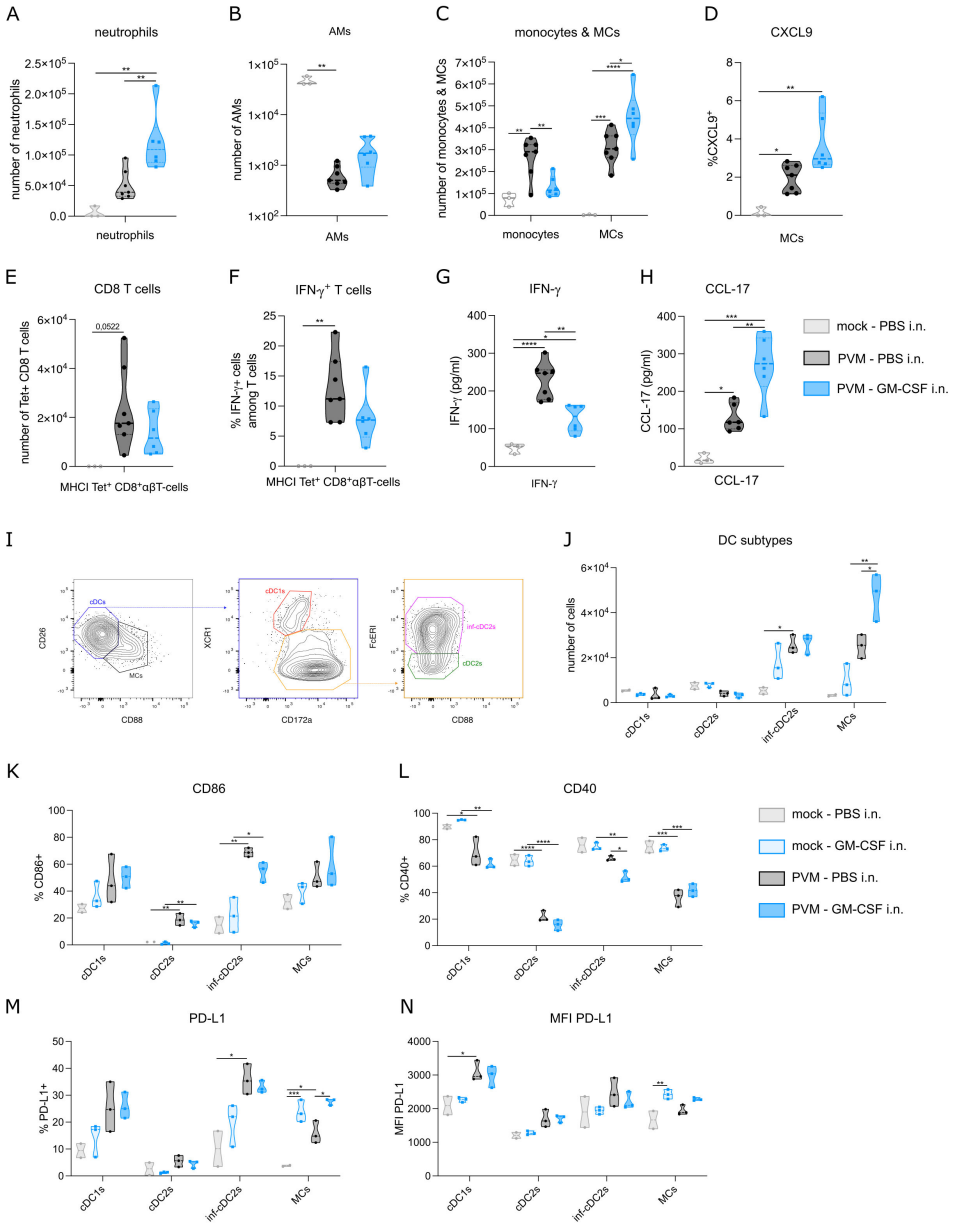
**(A)** Number of neutrophils in BAL fluid at 11 dpi. **(B, C)** Number of alveolar macrophages (AMs), monocytes and monocyte-derived cells (MCs) in lung at 11 dpi. **(D)** Intracellular CXCL9 expression in MCs from lung at 11 dpi. **(E)** Number of PVM-specific MHCI^+^ CD8 T cells in lung at 11 dpi. **(F)** Intracellular IFN-γ production in lung PVM-specific MHCI^+^ CD8 T cells at 11 dpi. **(G)** IFN-γ cytokine levels in BAL fluid at 11 dpi. **(H)** CCL-17 cytokine levels in BAL fluid at 11 dpi. **(I)** Gating strategy to distinguish MCs from cDCs, cDC1s from cDC2s and inflammatory cDC2s from non-inflammatory cDC2s. **(J)** Number of conventional DC1s (cDC1s), cDC2s, inflammatory cDC2s and MCs in lung on 8 dpi. **(K-N)** CD86, CD40 and PD-L1 expression on cDC subtypes and MCs. Data information: The presented data are from one representative experiment out of three independent repeats. All data were analyzed with a Shapiro-Wilk normality test to assess whether the data were normally distributed. Parametric data were analyzed with an ordinary one-way ANOVA test with multiple comparison correction. Nonparametric data were analyzed with an unpaired Kruskal-Wallis test with multiple correction. For the CCL-17 data, a Grubbs’ outlier test was performed and one statistical outlier was excluded from the PVM PBS group (p<0.05). Data are shown as means ± SEMs. *P <.05, **P <.01, ***P <.001 and ****P<.0001.

To quantify inflammation, we performed flow cytometry on lung and BAL fluid. As expected, neutrophils, monocytes, MCs and CD8+ T cells were strongly increased upon PVM infection, whereas AMs were reduced. Compared to PBS-treated PVM-infected mice, GM-CSF treated mice (as in **Figure 1J**) had an increased number of neutrophils in BAL fluid at 11 dpi (**Figure 2A**). This is in line with the myelopoietic and neutrophil-priming functions of GM-CSF (17). Along our hypothesis, we expected the number of AMs to be (partly) restored upon GM-CSF treatment, but there was only a slight and non-significant increase in AM numbers recovered (**Figure 2B**), even when higher concentrations of inhaled GM-CSF were used (data not shown). However, GM-CSF favored the differentiation of monocytes towards MCs, as reflected by a reduction in monocytes and their increased transition into monocyte-derived cells expressing CD11c and MHCII (**Figure 2C**). In inflammatory environments, recruited macrophages upregulate chemokine production and CXCL9 has been found as a good universal marker of recruited pro-inflammatory macrophages (18). However, there was only a trend towards more activation of MCs, as revealed by increased intracellular CXCL9 expression (**Figure 2D**). Given that T cells constitute the primary cell type at 11 dpi, we took a closer look at these cells by utilizing a tetramer stain to identify PVM-Nucleoprotein specific CD8 T cells (**Figure 2E**). The number and activation of tetramer+ CD8 T cells were not significantly altered upon GM-CSF treatment, although there was a trend towards less IFN-γ production (**Figure 2F**). Of note, IFN-γ concentration in BAL fluid was significantly reduced in GM-CSF treated groups at 11 dpi (**Figure 2G**). On the other hand, we could observe more CCL-17 production in BAL fluid, confirming a previously described GM-CSF – CCL-17 axis (**Figure 2H**) (19).

GM-CSF has been shown to play a cell-intrinsic pro-survival role in non-lymphoid conventional cDC1 dendritic cells (14). During PVM infection, inflammatory cDC2s (inf-cDC2s) are the main conventional DC type that infiltrates the lung (15). These inf-cDC2s induce both CD4 and CD8 T cells responses and their recruitment is not intrinsically dependent on GM-CSF (15). Inf-cDC2s share quite some surface markers with MCs, but they can be distinguished based on CD26 and CD88 expression (**Figure 2I**) (15, 20). Similar to the findings from Bosteels et al. (15), inf-cDC2s and MCs infiltrated the lung upon PVM infection at 8 dpi (**Figure 2J**). GM-CSF exposure elevated MC infiltration, but did not affect the number of inf-cDC2s. Co-stimulatory molecules such as CD86 were increased in PVM-infected mice compared to mock infection, but this was not modified further by inhaled GM-CSF administration (**Figure 2K**). Interestingly, CD40 expression was reduced on inf-cDC2s from GM-CSF-treated PVM mice compared to PBS-treated infected mice (**Figure 2L**). Expression of ligands for CD8 T cell checkpoint molecules such as PD-1 is typically seen in peripheral antigen presenting cells (APCs) in tissues. Expression of PD-L1 was induced by PVM infection in cDC1s and infl-cDC2s, but not in cDC2s, consistent with the idea that mainly cDC1s and infl-cDC2s have the capacity to stimulate CD8 T cells (**Figure 2M**). In MCs, PD-L1 was induced upon GM-CSF treatment, and this effect was also seen in PVM-infected mice, which attained the highest percentage of MCs expressing PD-L1. When studying the intensity of PD-L1 expression, it was clear that mainly cDC1s attained the highest PD-L1 expression (**Figure 2N**). GM-CSF given in the absence of viral infection only induced PD-L1 expression on MCs. Thus, activation of the most prevalent DC subtypes upon PVM infection was altered after inhaled GM-CSF treatment.

### CD8 T cell proliferation is reduced in presence of local GM-CSF

**Figure 3:**
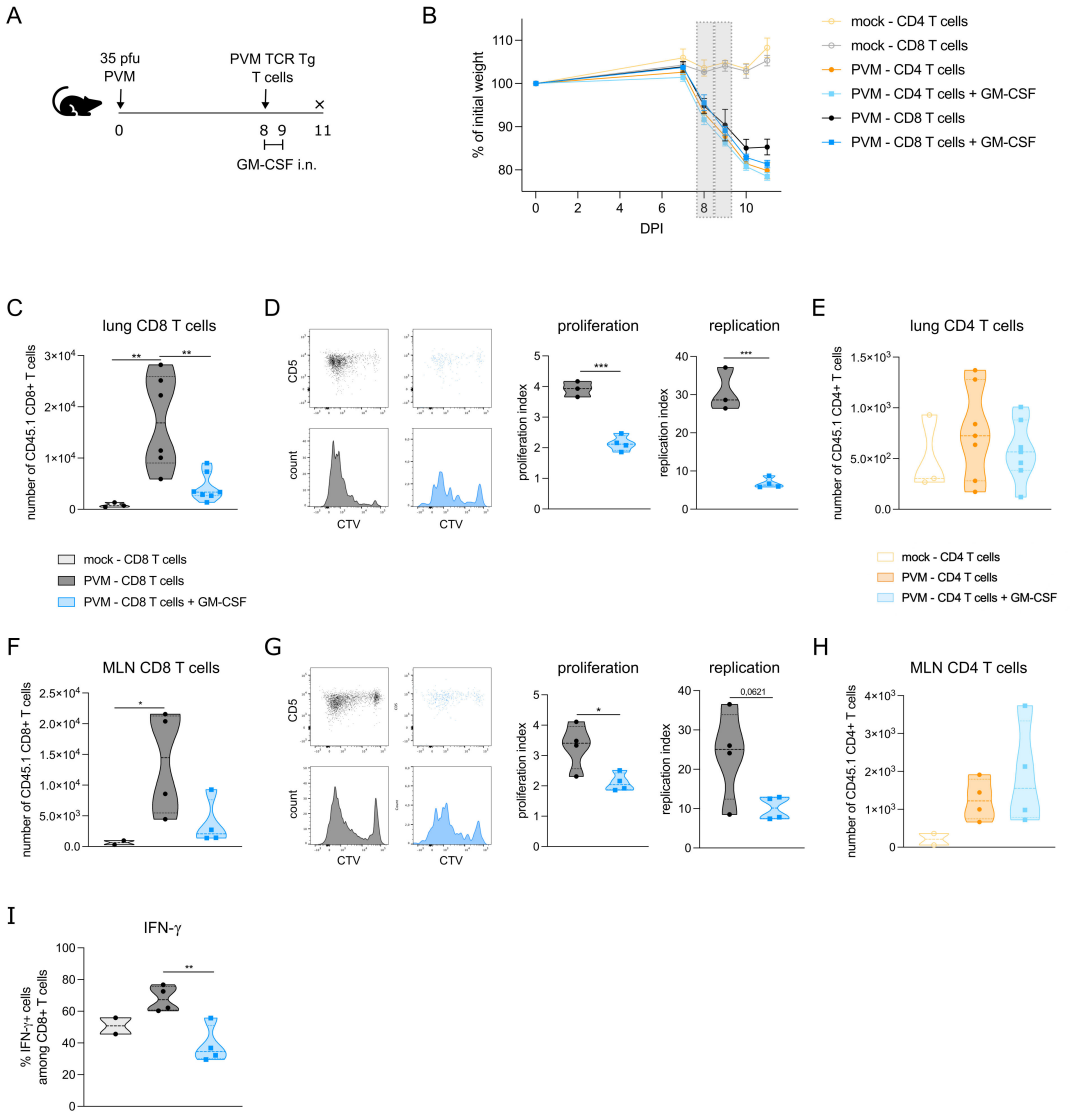
**(A)** Schematic set-up of the experiment. **(B)** Weight curves of all groups in the experiment. The grey bars represent the days of GM-CSF treatment. **(C)** Number of transgenic CD45.1 CD8^+^ T cells in lungs of recipient mice. **(D)** CTV proliferation profile of transgenic CD45.1 CD8^+^ T cells in lungs of recipient mice and quantified by the proliferation and replication index. **(E)** Number of transgenic CD45.1 CD4^+^ T cells in lungs of recipient mice. **(F)** Number of transgenic CD45.1 CD8^+^ T cells in mediastinal lymph node (MLN) of recipient mice. **(G)** CTV proliferation profile of transgenic CD45.1 CD8^+^ T cells in MLN of recipient mice and quantified by the proliferation and replication index. **(H)** Number of transgenic CD45.1 CD4^+^ T cells in MLN of recipient mice. In D and G, mock mice are not shown due to the lack of a transgenic T cell population. **(I)** Intracellular IFN-γ production in lung PVM TCR Tg CD8 T cells. Data information: The presented data are from one representative experiment out of two independent repeats. All data were analyzed with a Shapiro-Wilk normality test to assess whether the data were normally distributed. Parametric data were analyzed with an ordinary one-way ANOVA test with multiple comparison correction or Students’ t-test. Nonparametric data were analyzed with an unpaired Kruskal-Wallis test with multiple correction or Mann-Whitney test. Data are shown as means ± SEMs. *P <.05, **P <.01 and ***P <.001.

The observed effects of GM-CSF on APC costimulatory and checkpoint ligands made us wonder whether T cell proliferation would be altered upon GM-CSF exposure. To address this question, we made use of PVM TCR transgenic mice that express either PVM_M37-47 specific CD4^+^ T cells or N339–347 specific CD8^+^ T cells. CTV-labelled CD4 or CD8 T cells of these CD45.1^+^ transgenic mice were adoptively transferred into PVM-infected CD45.2^+^ recipient mice at 8 dpi (**Figure 3A**). Two and 24 hours after T cell transfer, mice were exposed to 5 μg GM-CSF or PBS intranasally. The adoptive T cell transfer did not change the PVM-induced weight loss in the recipient mice (**Figure 3B**), suggesting that T cells were not contributing to disease severity in this model. On 11 dpi, PVM TCR Tg T cell proliferation was measured in lymph nodes and lung. Both in lung and mediastinal lymph nodes, PVM TCR Tg CD8 T cells were reduced upon GM-CSF exposure (**Figures 3C, F**), whereas the number of CD4 T cells did not differ (**Figures 3E, H**). Upon analysis of their CTV-dilution profile, we found that the transgenic CD8 T cells had proliferated less in the GM-CSF treated group (**Figure 3D, G**). Furthermore, these GM-CSF exposed CD8 T cells showed less IFN-γ production in the lung (**Figure 3I**), indicative of a lower activation state.

## Discussion

In respiratory virus infections, such as influenza and SARS-CoV-2, local GM-CSF administration has been shown to have a beneficial effect. For instance, in SARS-CoV-2 infected patients, inhaled GM-CSF supported the homeostatic AM pool (7) and resulted in more activated IFN-γ^+^ CD8 T cells. In mouse models of influenza, the protective effect of GM-CSF against viral-induced weight loss could be attributed to AMs and CD103^+^ DCs (11–13). Important to note is that GM-CSF was given in a prophylactic manner in the influenza studies, and not in a therapeutic setting in mice with active viral replication already underway. Here, to be clinically translational, we administered GM-CSF locally during ongoing infection. We used a PVM infection model, which recapitulates the disease pathology of RSV in humans. In contrast to influenza, the immunopathology caused by PVM is not caused by neutrophils, but rather is thought to result from CD8 T cells producing IFN-γ (21–24). In general, antigen presentation to CD8^+^ T cells is attributed to cDC1s, which excel in their cross-presenting capacity. Interestingly, this cross-presenting capacity can be enhanced by and is dependent on GM-CSF (14, 25). However, in inflammatory conditions such as viral infection, inflammatory cDC2s are the main infiltrating DC subtype that are capable to prime CD8^+^ T cells. These inflammatory cDC2s are not intrinsically dependent on GM-CSF (15).

GM-CSF therapy during PVM infection did not improve clinical signs of disease, although there were changes in the lung immunome. Of note, we also performed experiments with anti-GM-CSF antibodies during ongoing infection, which did not impact disease severity either. Beside an increase in neutrophils, GM-CSF treatment favored the transition of monocytes towards MCs. We hypothesize that this might be attributed to IRF5 activation. A GM-CSF-IRF5 axis has been shown in several cell types (26–28) and IRF5 activation guides monocytes toward an inflammatory macrophage phenotype, producing chemokines (29). There might be various reasons why PVM-specific CD8 T cell responses, and not CD4 T cells responses, were reduced in presence of GM-CSF. Given that T cells do not express the GM-CSF receptor, the inhibitory effect on T cells should be indirect, most probably via antigen presenting cells. The specific effect on CD8 T cells would suggest that the function of cDC1s or inf-cDC2s would be altered in presence of GM-CSF. Indeed, the level of CD40 and CD86 is lower on inf-cDC2s of GM-CSF treated mice. GM-CSF is also an inducer of myeloid suppressor cells, which inhibit IFN-γ^+^ producing cells (30–32). In this way, reduced T cell activation in presence of GM-CSF has been shown in several cancer models (33–35). The observed GM-CSF dependent increase in PD-L1 expression on MCs from PVM infected mice has also been seen on tumor-associated macrophages (36, 37) and myeloid suppressor cells (30), which might also contribute to the reduced CD8 T cell activation (38). Lastly, CCL-17 in BAL fluid was increased by GM-CSF, confirming the previously described GM-CSF – CCL-17 axis (19). As CCL-17, by binding to its CCR4 receptor (39), is an attractant for regulatory T cells, the reduced effector T cell responses might also be attributed to regulatory T cells. In fact, additional experiments should be performed to address whether these hypotheses apply to our findings. However, the observed effects on T cells, as well as on myeloid cells, did not result in an altered clinical outcome during PVM infection. As such, inhaled GM-CSF therapy cannot be considered as a universal protective therapy in respiratory virus infections.

## Data Availability

The raw data supporting the conclusions of this article will be made available by the authors, without undue reservation.
